# Blood Inflammatory Markers and Cytokines in COVID‐19 Patients With Bacterial Coinfections

**DOI:** 10.1002/iid3.70105

**Published:** 2024-12-18

**Authors:** Qingqing Bi, Jie Zhu, Jinju Zheng, Qingyun Xu, Juan Chen, Lei Zhang, Xiaofeng Mu

**Affiliations:** ^1^ Department of Laboratory Medicine Qingdao Central Hospital Qingdao China; ^2^ Department of Clinical Laboratory Peking University First Hospital Beijing China

**Keywords:** bacterial coinfection, COVID‐19, cytokines, inflammatory markers

## Abstract

**Background:**

Bacterial coinfection in patients with SARS‐CoV‐2 infection is an important risk factor for death. This study investigated whether there were differences in levels of serum inflammatory markers in COVID‐19 patients with bacterial coinfections compared with those without bacterial infection.

**Methods:**

A total of 235 inpatients with SARS‐CoV‐2 infection admitted to Qingdao Central Hospital from December 7, 2022, to August 7, 2024, were included. Patients were divided into a bacteria‐positive group (115 cases) and a bacteria‐negative group (120 cases) according to whether they had bacterial coinfections. PCT, CRP, and 12 kinds of cytokines were compared between groups, and the distribution of bacterial species in the positive group was statistically analyzed.

**Results:**

The serum levels of CRP (*Z* = 8.94, *p* < 0.001), PCT (*Z* = 5.59, *p* < 0.001), IL‐1β (*t* = 4.863, *p* < 0.001), IL‐2 (*t* = 5.810, *p* < 0.001), IL‐5 (*t* = 3.837, *p* < 0.001), IL‐6 (*t* = 4.910, *p* < 0.001), IL‐8 (*t* = 3.325, *p* < 0.001), ILIL‐12p70 (*t* = 4.722, *p* < 0.001), IL‐17 (*t* = 3.315, *p* = 0.001) and TNF‐α (*t* = 4.251, *p* < 0.001) between the two groups were significantly different. IL‐4, IL‐10, IFN‐α, and IFN‐γ were not statistically significant (*p* > 0.05). Among the 115 bacteria‐positive patients, 56 patients were positive for one species and 59 patients were multiple infections. *Acinetobacter baumannii*, *Klebsiella pneumoniae, Pseudomonas aeruginosa*, *Staphylococcus aureus,* and *Haemophilus influenzae* were common species.

**Conclusions:**

Serum PCT and CRP levels in COVID‐19 patients with bacterial coinfection are higher than those without bacterial infection. Cytokines such as IL‐1β, IL‐2, IL‐5, IL‐6, IL‐8, IL‐12p70, IL‐17, and TNF‐α may be involved in the progression of COVID‐19 combined with bacterial infection. They can be used as potential markers to evaluate the disease condition and prognosis.

## Graphical abstract

Serum cytokines including IL‐1β, IL‐2, IL‐5, IL‐6, IL‐8, IL‐12 p70, IL‐17, and TNF‐α were significantly higher in COVID‐19 patients with bacterial coinfections than those without bacterial infection. These cytokines may be involved in the progression of COVID‐19 combined with bacterial coinfections.

## 1 Introduction

The global pandemic of COVID‐19 caused by SARS‐CoV‐2 poses an unprecedented challenge to our healthcare system. The novel coronavirus spreading worldwide is a single‐stranded RNA virus in the coronavirus family that mainly infects the respiratory and intestinal tracts, causing various symptoms [1]. The virus has a variety of transmission routes, strong infectivity, different incubation periods, early clinical symptoms are not obvious, the development of severe and critical cases is rapid, and the fatality rate is high [2]. Although most COVID‐19 patients only have mild or moderate symptoms, some patients will develop severe pneumonia and even ARDS requiring mechanical ventilation [3]. It is well known that various bacteria can colonize and, under appropriate conditions, cause infection throughout the respiratory tract. It is now widely accepted that in all influenza pandemics of the last century, secondary or concurrent bacterial coinfection was the leading cause of death. Both bacterial and viral coinfection of the respiratory tract can be secondary or simultaneous. Multiple synergistic interactions between viruses and bacteria have been shown. Secondary and concurrent bacterial and viral coinfections with other viral respiratory pathogens have been thoroughly studied. However, studies on SARS‐CoV‐2 coinfections with bacteria and antibiotic resistance issues are still limited.

Bacterial infection of the body induces the production of a variety of cytokines, chemokines, and acute chronotropic response proteins, such as IL‐6, IL‐10, C‐reactive protein (CRP), and procalcitonin (PCT), by the body's immune cells. Immune function is a strong defense against invasive pathogens. The active immune response in the body is described as a cytokine storm, which is usually clinically manifested by a systemic inflammatory response, MODS, and a rapid and substantial increase in inflammatory marker levels [4]. Cytokine storms occurring in COVID‐19 patients are associated with extensive tissue damage in vivo [5], including ARDS. CRP and PCT can be used in the auxiliary diagnosis of bloodstream bacterial infection. IL‐6 is mainly produced by macrophages, T cells, B cells, and other cells, which regulate the immune response and acute phase reaction and play an essential role in the body's immune response against infection [6]. As one of the cellular inflammatory markers, the level of IL‐6 is low in healthy people and increases with the occurrence of bacterial infections. The higher the degree of disease, the higher the content will be. IL‐10 is a cytokine secreted by a subpopulation of regulatory T cells, mainly by Th2 cells. In addition to mediating the immune response, the main biological activity of IL‐10 is immunosuppression. IL‐17, produced primarily in peripheral blood T cells, is one of the most critical proinflammatory markers in the body and is involved in several inflammatory responses [7].

Secondary bacterial infection following novel coronavirus infection is a significant risk factor for death, and bacterial infection can further exacerbate the patient's inflammatory response, leading to multiple organ failures. According to a review article on coinfections in several hospitals, 62/806 (8%) patients with COVID‐19 developed bacterial infections during hospitalization [8]. Another study showed that 102 of 1125 patients (9.1%) with COVID‐19 were diagnosed with secondary bacterial pneumonia [9]. There are limitations in the diagnosis of bacterial coinfections in patients with coronavirus infection, and the patient's condition is usually relatively serious when diagnosed. Therefore, how to assess bacterial infection in an early stage has important clinical significance.

The aim of this study was to analyze the levels of blood inflammation‐related factors in patients with bacterial coinfection and compare them with those without bacterial infection. It is hoped that these findings can provide references for the early assessment of coinfected patients and aid clinical diagnosis and treatment decisions.

## 2 Methods

### 2.1 Study Design and Patients

A total of 2246 inpatients with COVID‐19, including 1415 males and 831 females aged between 23 and 98, were admitted to Qingdao Central Hospital between December 7, 2022, and August 7, 2024. Medical records and general laboratory test results were collected from the hospital information management system.

Inclusion criteria: SARS‐CoV‐2 nucleic acid positive; lower respiratory specimens (sputum or bronchoalveolar lavage fluid, BALF) were sent for bacterial culture and identification. Exclusion criteria: serious diseases of the blood or immune system; pregnancy, lactation, malignant tumor, and other special groups; positive bacterial reports preceded positive SARS‐CoV‐2 nucleic acid; complicated by additional viral, mycoplasma, or other infectious diseases.

After exclusion, 235 COVID‐19 patients were tested for bacterial nucleic acid, bacterial culture, serum cytokines, and other inflammatory factors at the same time, of which 115 were positive for bacterial nucleic acid and culture, and 120 were negative. They were divided into two groups: bacteria‐positive and bacteria‐negative groups.

This study was approved by the Research Management Department of Qingdao Central Hospital. Informed consent was acquired upon admission and ethics approval was not necessary as it was considered a retrospective analysis of the treatment and care already provided.

### 2.2 Laboratory Tests

All sample collection, temporary storage, and cold chain transfer procedures are strictly in accordance with detection SOP and kit instructions. Upper respiratory specimens (nasopharyngeal and oropharyngeal swabs were collected for SARS‐CoV‐2 detection. Lower respiratory specimens (deep sputum or BALF specimens) were collected for bacteria detection and culture. Blood samples were collected for bacteria culture. Serum samples were used for inflammatory factors detection. All bacterial tests were performed in a biosafety level 2 laboratory.

SARS‐CoV‐2 nucleic acids were detected using TaqMan real‐time PCR assay (Daan Gene, Guangzhou, China). Bacteria nucleic acids were detected using Loop‐mediated isothermal amplification (Capitalbio, Beijing, China). After adding the extracted nucleic acid to a microfluidic chip, real‐time fluorescence analysis was performed on the constant temperature amplification nucleic acid analyzer at 65°C.

Bacterial culture and identification were performed by qualified clinical microbiologists. Specimen preparations: Sputum specimens: Wash the sputum with sterile saline or add an equal amount of 1% trypsin solution to homogenize the sputum and prepare it for use. BALF specimens: Mix the BALF specimen by vortexing vigorously for 30–60 s. Subsequently, they were inoculated onto the sputum device (blood agar plate, chocolate agar plate, and MacConkey agar plate) and incubated at 35°C in a 5%–10% CO_2_ environment for 18–24 h. Based on the colony and morphological characteristics, a preliminary judgment was made and then the bacterial characteristics were identified by biochemical identification.

PCT levels were detected using an AIA2000 immunoluminescence analyzer. PCT > 0.50 ng/mL was considered positive. Neutrophils and lymphocytes were determined using a Sysmex XN2800 blood cell analyzer. CRP was determined using an Aristo‐specific protein analyzer; CRP > 5.000 mg/L was considered positive. Cytokine detections were performed on flow cytometry using a commercial kit that simultaneously detects 12 cytokines. For the bacteria‐positive group, all inflammatory factor levels were measured during bacterial coinfection positive.

### 2.3 Statistical Analysis

Data were summarized using EXCEL 2019, and SPSS 25.0 statistical software was applied to analyze the data. Qualitative data were expressed as numbers (%), using the chi‐square test. Quantitative data with a normal distribution were expressed as *x* ± *s*, and an independent samples *t*‐test was used for comparisons between the two groups. Quantitative data with nonnormal distribution were expressed by M (P25, P75), and the rank sum test was used for comparison between the two groups. Multiple groups of quantitative data conforming to a normal distribution are expressed by *x* ± *s*, and the *F* test was used for comparison between groups. *p* < 0.05 was considered a statistically significant difference.

## 3 Results

### 3.1 Study and Sample Selection

**Figure 1:**
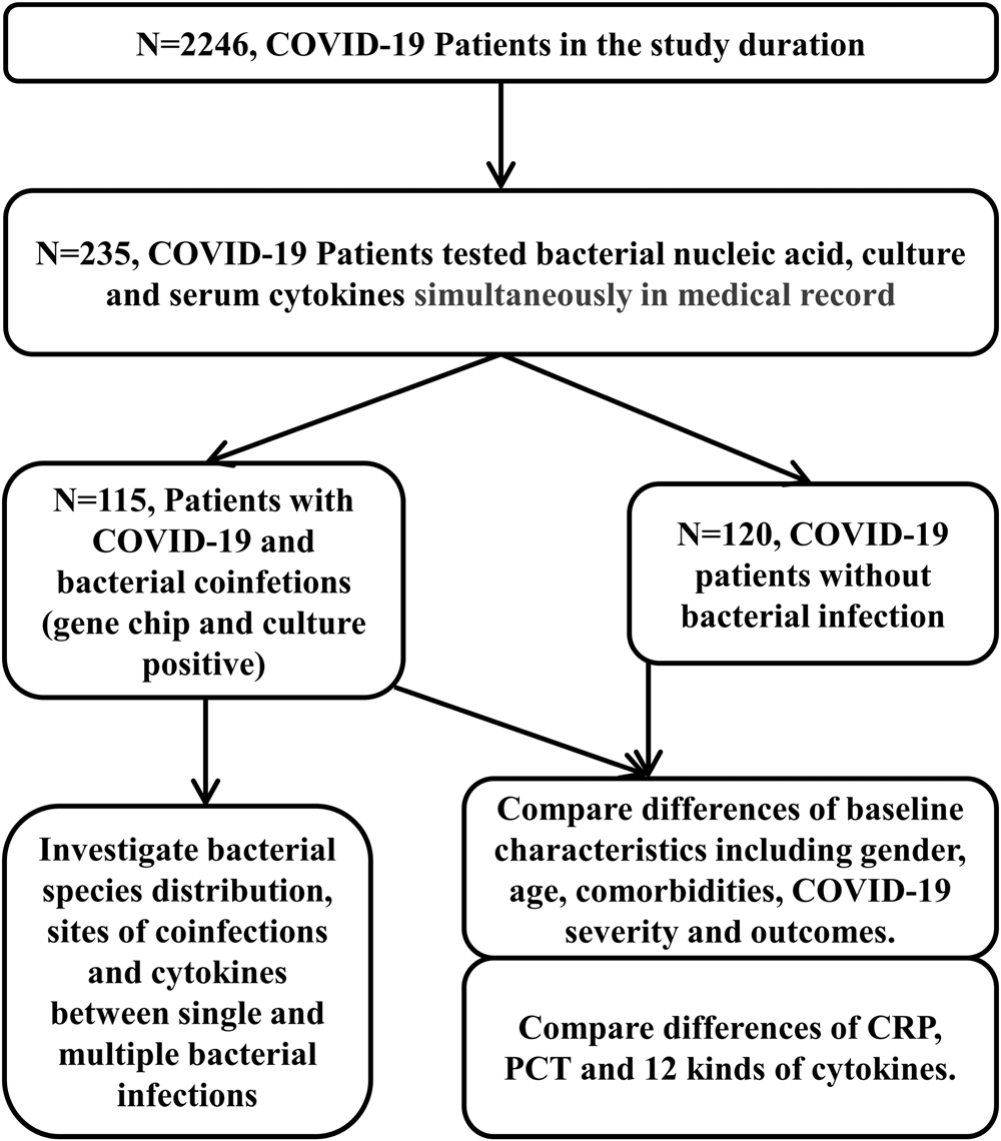
Study design, sample selection, and comparison process.

As shown in Figure 1, 235 COVID‐19 patients were tested for bacterial nucleic acid, bacterial culture, serum cytokines, and other inflammatory factors at the same time, of which 115 were positive for bacterial nucleic acid and culture, and 120 were negative. They were divided into two groups: bacteria‐positive and bacteria‐negative groups.

### 3.2 Comparison of Baseline Characteristics Between Bacteria‐Positive and Bacteria‐Negative Groups

As presented in Table 1, in the bacterial‐positive group, there were 75 (65.2%) male and 40 (34.8%) female cases out of a total of 115 cases, with a mean age of 74.20 ± 11.48 years. In the bacterial‐negative group, there were 82 (68.3%) male and 38 (31.7%) female cases out of a total of 120 cases, with a mean age of 71.80 ± 9.02 years. Differences in mean gender, age, and comorbidities were not statistically significant between the two groups (*X* ^2^ = 0.257, *t* = 1.786, *X* ^2^ = 0.211, all *p* > 0.05). COVID‐19 severity and patient outcomes were statistically significant between the two groups (*t* = 2.369, *X* ^2^ = 20.71, both *p* < 0.05).

**Table 1:** Baseline characteristics of bacteria‐positive and bacteria‐negative groups.

Characteristics	Bacteria positive *N* = 115	Bacteria negative *N* = 120	Differences	
Gender, *N* (%)			Chi‐square test	
Male	75 (65.2)	82 (68.3)	*X* ^2^ values	*p*
Female	40 (34.8)	38 (31.7)	0.257	0.612
Age (years, *x* ± *s*)	74.20 ± 11.48	71.80 ± 9.02	*t*	*p*
			1.786	0.075
Comorbidities, *N*			Chi‐square test	
CKD	21	15	*X* ^2^ values	*p*
Hypertension	43	37	0.211	0.646
Type 2 DM	29	22		
COVID‐19 severity, *N* (%)			*t*	*p*
Mild	30 (26.1)	44 (36.7)	2.369	0.019
Moderate	48 (41.7)	52 (43.3)		
Severe	31 (27.0)	21 (17.5)		
Critical	6 (5.2)	3 (2.5)		
Outcome, *N* (%)			Chi‐square test	
Non‐survivor	25 (21.7)	3 (2.5)	*X* ^2^ values	*p*
Survivor	90 (78.3)	117 (97.5)	20.71	< 0.001

Abbreviations: CKD, chronic kidney disease; DM, diabetes mellitus.

### 3.3 Comparison of CRP and PCT Levels Between Bacteria‐Positive and Bacteria‐Negative Groups

Among the 235 SARS‐CoV‐2‐positive cases, the median CRP level was 78.20 (26.16, 137.20), and the median PCT level was 0.80 (0.17, 2.45) in the bacteria‐positive group. In contrast, the median CRP level was 9.15 (2.74, 31.77), and the median PCT level was 0.23 (0.08, 0.59) in the bacteria‐negative group. CRP and PCT levels were higher in the positive group, and both were significantly different between the two groups (*Z* = 8.94, *p* < 0.001; *Z* = 5.59, *p* < 0.001) (Table 2).

**Table 2:** Comparison of CRP and PCT between bacteria‐positive and bacteria‐negative groups.

Groups		M (P_25_,P_75_)	95% CI of median difference value	Wilcoxon rank sum test	
				*Z* value	*p* value
CRP	Bacteria positive	78.20 (26.16, 137.20)	78.22–104.50	8.94	< 0.001
	Bacteria negative	9.15 (2.74, 31.77)	15.83–28.74		
PCT	Bacteria positive	0.80 (0.17, 2.45)	1.85–3.63	5.59	< 0.001
	Bacteria negative	0.23 (0.08, 0.59)	0.32–0.56		

### 3.4 Comparison of Cytokines Between Bacteria‐Positive and Bacteria‐Negative Groups

**Figure 2:**
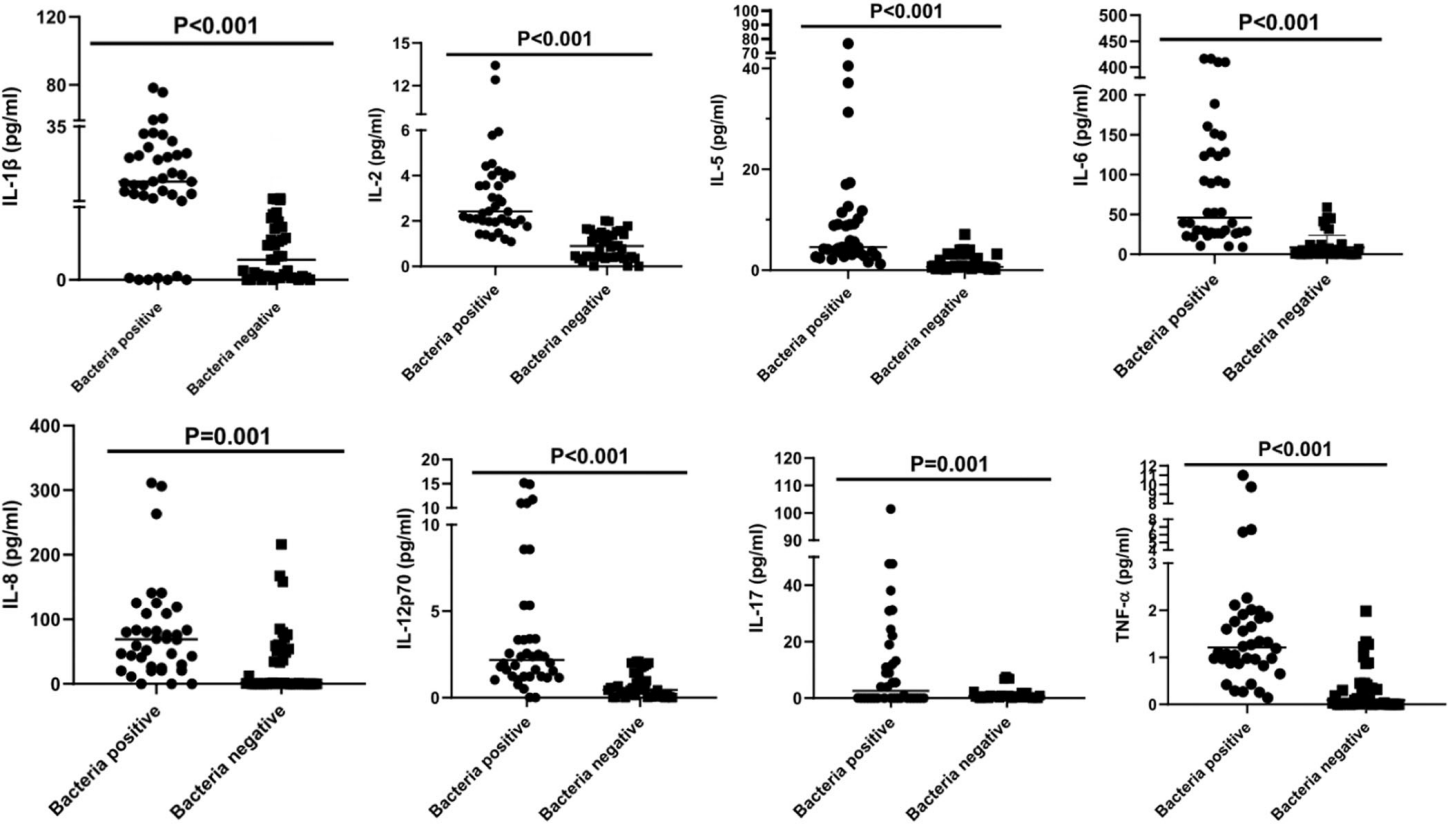
Scatter plot of cytokines with significant differences between bacteria‐positive and bacteria‐negative groups.

As presented in Table 3, the bacteria‐positive group had significantly higher interleukin 1β (difference 16.12, *t* = 4.863, *p *< 0.001), interleukin 2 (difference 2.42, *t* = 5.810, *p* < 0.001), interleukin 5 (difference 8.68, *t* = 3.837, *p* < 0.001), interleukin 6 (difference 91.99, *t* = 4.910, *p* < 0.001), interleukin 8 (difference 47.63, *t* = 3.325, *p* < 0.001), interleukin 12p70 (difference 3.05, *t* = 4.722, *p* < 0.001), interleukin 17 (difference 10.54, *t* = 3.315, *p* = 0.001) and TNF‐α (difference 1.63, *t* = 4.251, *p* < 0.001) levels than the bacteria‐negative group (*p* < 0.05). There was no significant difference in interleukin 4 (difference 0.42, *t* = 0.767, *p* = 0.446), interleukin 10 (difference 7.87, *t* = 0.378, *p* = 0.707), α‐interferon (difference 0.81, *t* = 0.998, *p* = 0.322), and γ‐interferon (difference 1.87, *t* = 1.844, *p* = 0.069) between the two groups. In addition, Figure 2 shows the eight cytokines with significant differences.

**Table 3:** Comparison of cytokines between bacteria‐positive and bacteria‐negative groups.

Cytokines	x ± s		*t‐*test	
	Bacteria positive	Bacteria negative	*t* value	*p* value
Interleukin 1β	17.37 ± 20.580	1.25 ± 5.05	4.863	< 0.001
Interleukin 2	3.31 ± 2.60	0.89 ± 0.58	5.810	< 0.001
Interleukin 4	1.70 ± 1.98	1.28 ± 2.80	0.767	0.446
Interleukin 5	10.32 ± 14.39	1.64 ± 1.61	3.837	< 0.001
Interleukin 6	100.49 ± 118.96	8.50 ± 15.26	4.910	< 0.001
Interleukin 8	79.62 ± 74.86	31.98 ± 51.05	3.325	0.001
Interleukin 10	29.69 ± 91.16	21.82 ± 93.90	0.378	0.707
Interleukin 12p70	3.76 ± 4.07	0.70 ± 0.71	4.722	< 0.001
Interleukin 17	11.78 ± 20.26	1.24 ± 2.02	3.315	0.001
α‐interferon	3.98 ± 2.69	3.16 ± 4.29	0.998	0.322
γ‐interferon	5.60 ± 4.65	3.73 ± 4.36	1.844	0.069
TNF‐α	1.94 ± 2.41	0.30 ± 0.46	4.251	< 0.001

### 3.5 Bacterial Species Distribution in the Positive Group

**Figure 3:**
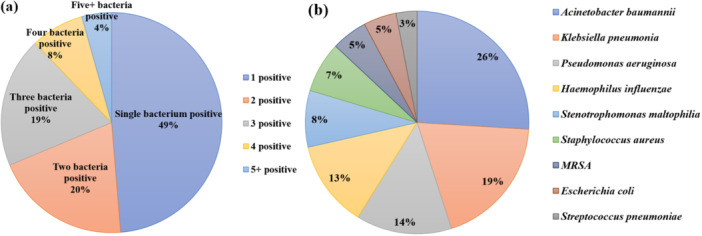
(a) Percentage of bacteria single and multiple coinfections. (b) Bacteria species distribution in the positive group.

As shown in Figure 3, among the 115 bacteria‐positive patients, 56 patients were single bacterium positive, with *Acinetobacter baumannii, Klebsiella pneumoniae,* and *Pseudomonas aeruginosa* being common. Twenty‐three patients were positive for two species of bacteria, with *A. baumannii* combined with *P. aeruginosa* being common. Twenty‐two patients were positive for three species of bacteria, and nine patients were positive for four species of bacteria. In addition, the top three positive species were *A. baumannii, K. pneumoniae,* and *P. aeruginosa*.

### 3.6 Comparison of Cytokines Between Single and Multiple Bacterial Coinfections

Focusing on COVID‐19 patients with bacteria coinfection, the levels of cytokines were tested between single and multiple bacterial coinfections by independent samples *t*‐test. As presented in Table 4, except for interleukin‐6, the remaining cytokines did not differ significantly between single and multiple bacterial coinfections. Interleukin‐6 was significantly different between single and multiple bacterial coinfections (difference 101.10, *t* = 2.863, *p* = 0.007).

**Table 4:** Comparison of cytokines between single and multiple bacterial coinfections.

Cytokines	*x* ± *s*		*t‐*test	
	Single bacterial infection	Multiple bacterial infection	*t* value	*p* value
IL‐1β	15.98 ± 20.87	18.76 ± 20.75	0.412	0.683
IL‐2	3.38 ± 2.72	3.25 ± 2.54	0.161	0.873
IL‐4	1.88 ± 2.51	1.53 ± 1.33	0.547	0.588
IL‐5	11.45 ± 18.15	9.19 ± 9.71	0.478	0.635
IL‐6	151.04 ± 147.60	49.94 ± 43.71	2.863	0.007
IL‐8	86.69 ± 80.31	72.55 ± 70.58	0.577	0.568
IL‐10	30.01 ± 92.33	29.38 ± 92.50	0.021	0.983
IL‐12p70	4.47 ± 4.38	3.04 ± 3.71	1.091	0.283
IL‐17	12.01 ± 24.97	11.55 ± 14.83	0.069	0.946
α‐interferon	4.37 ± 2.93	3.59 ± 2.44	0.892	0.378
γ‐interferon	6.42 ± 5.78	4.78 ± 3.11	1.084	0.286
TNF‐α	1.94 ± 2.34	1.93 ± 2.54	0.014	0.989

### 3.7 Bacterial Species Distributions in Respiratory Tract and Bloodstream Infections

**Figure 4:**
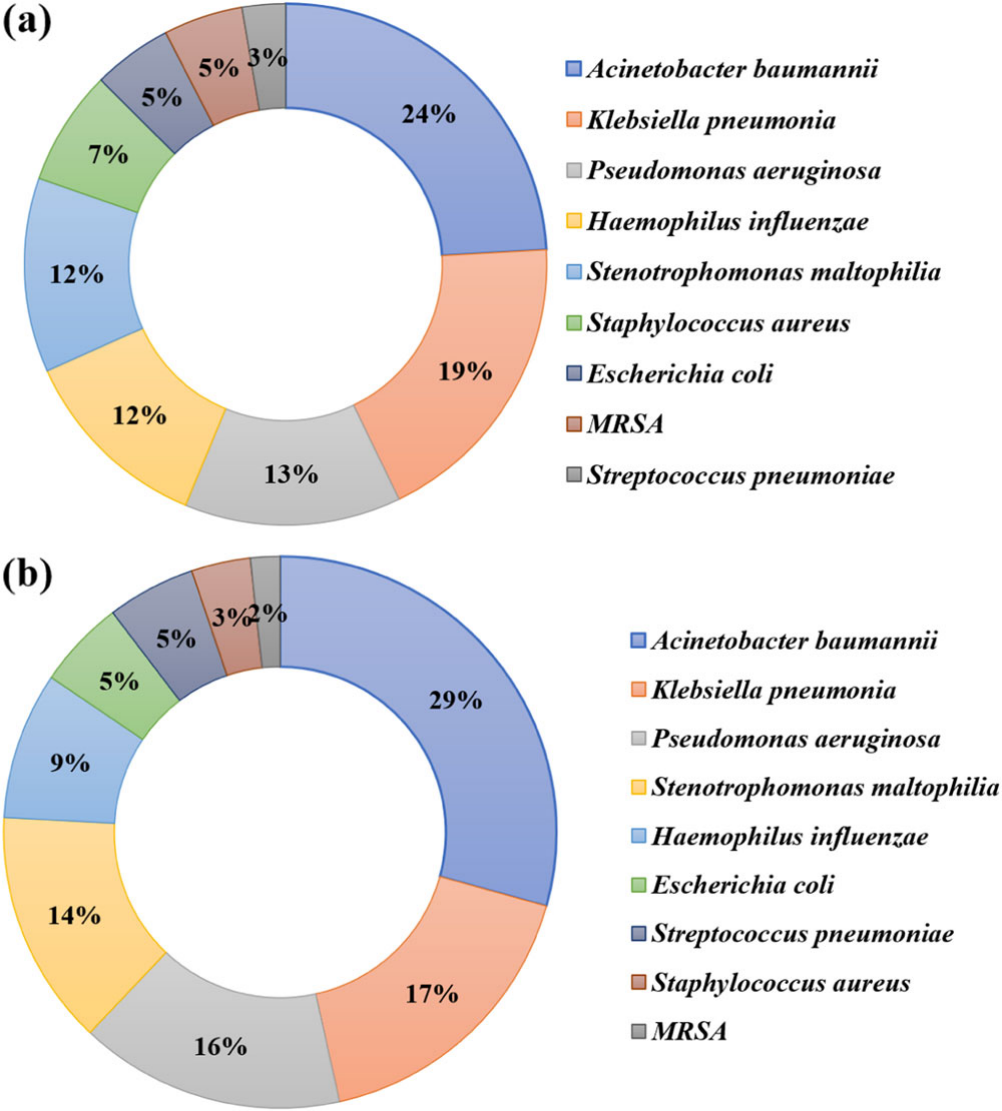
Distribution of species within respiratory tract infection (a) and bloodstream infection (b).

Bacteria‐positive patients were classified by sites of infection, including 109 cases of respiratory tract infection, 20 cases of bloodstream infection, 13 cases of respiratory tract and bloodstream coinfection, and 4 cases of respiratory tract and urinary tract coinfection. The distribution of species within respiratory tract infection (Figure 4a) and bloodstream infection (Figure 4b) was counted. The top three positive species were *A. baumannii*, *K. pneumonia,* and *P. aeruginosa*. Further, antibiotic resistance of these coinfection bacteria was investigated and available in Table S1. Carbapenem‐resistant *A. baumannii*, *K. pneumonia,* and *P. aeruginosa* were common antibiotic‐resistant phenotypes.

## 4 Discussion

COVID‐19 combined with bacterial coinfection refers to bacterial infection occurring during the period of SARS‐CoV‐2 infection according to microbial reports, serological tests, and the clinical judgment of doctors [10]. The reported incidence of bacterial coinfection ranges from 3% to 30%, with a higher incidence in the elderly [11]. Some retrospective studies found that old age and underlying medical conditions were the main risk factors, while other risk factors included immunosuppressant use, glucocorticoid use, indwelling urinary catheters, and ventilator use. Severe cases are more likely to develop bacterial infections, and COVID‐19 patients with bacterial infections have a worse prognosis and higher mortality [12].

Many studies reported that COVID‐19 patients with inflammatory factors higher than normal cutoff values had significantly more frequent coinfections, and a higher risk of mortality compared to COVID‐19 patients with lower inflammatory factor levels [12]. This study focused on the differences in inflammatory factors in bacteria‐positive and bacteria‐negative COVID‐19 patients. It analyzed the differences between 12 kinds of cytokines, providing some new data reference for the clinical management of COVID‐19 patients with bacteria coinfections.

CRP is the most common marker of inflammation. It can be used as an important marker for the diagnosis of infectious diseases. The current CRP assay is diverse, rapid, and inexpensive, so the CRP concentration is used in a regular manner for the diagnosis, differential diagnosis, and prognosis of SARS‐CoV‐2 infection in the management of COVID‐19 [13]. In this study, the CRP concentration in the bacteria‐positive group was significantly higher than that in the bacteria‐negative group. PCT, one of the precursors of calcitonin, is produced by thyroid C cells, and PCT expression is not elevated or is slightly increased when viral infections occur. However, its expression is significantly increased only in severe systemic infections [14]. A systematic review also indicated that PCT was a tool for antimicrobial stewardship in COVID‐19 patients with bacterial coinfections [15]. In this study, the serum PCT level of the bacteria‐positive group was significantly higher than that of the bacteria‐negative group, and the serum PCT level of the COVID‐19 patients without bacterial coinfection only increased slightly, indicating that detection of the PCT level of COVID‐19 patients can assist in diagnosing whether there is secondary bacterial infection after viral infection and may also quickly identify the severity of their infection to help determine whether antibiotics are needed to prevent sepsis. The increase in CRP and PCT levels was significant and disease progression of COVID‐19 patients with bacterial coinfection was faster, all of which suggested that bacterial coinfection could cause disease aggravation.

The mechanism of injury caused by SARS‐CoV‐2 infection is the interaction between the virus and the body's immune system. Cellular damage caused directly by viruses is only one of the pathogenic mechanisms, while the cytokine storm induced by an excessive inflammatory response and immune homeostasis imbalance after infection and injury is a key factor leading to severe illness and even death [16]. Most individuals infected with SARS‐CoV‐2 clear the infection in the upper respiratory tract, presenting asymptomatic to mild disease. When this initial response is inadequate, SARS‐CoV‐2 narrowly migrates into the lower airways, resulting in moderate or severe disease characterized by acute respiratory distress syndrome, pneumonia, and cytokine storms. Studies have shown that cytokine storm production is also a pathogenic mechanism in COVID‐19 and correlates with disease severity [17]. Cytokine storms are triggered by the excessive and uncontrolled release of proinflammatory cytokines due to the massive and rapid replication of the virus, where the body's immune system is overactivated and the immune balance is disturbed [18, 19]. Clinical studies have also shown that serum levels of proinflammatory cytokines (IFN‐γ, IL‐1β, IL‐6, IL‐12, and TGF‐β) and chemokines (CCL2, CXCL10, CXCL8, and CXCL9) are elevated in patients with severe disease compared to those without severe disease, while levels of the anti‐inflammatory cytokine IL‐10 are very low [20].

In this study, proinflammatory cytokines including IL‐1β, IL‐2, IL‐5, IL‐6, IL‐8, IL‐12p70, IL‐17, and TNF alpha were all significantly higher in COVID‐19 patients in the bacteria‐positive group than in the bacteria‐negative group, while there were no significant differences in IL‐4 and IL‐10 levels. IL‐1β, as an important immunomodulatory factor, can produce chemotactic effects on neutrophils and activate B cells to produce protective antibodies. If stress reactions such as burns and sepsis occur, IL‐1β secretion can be abnormally elevated and positively correlated with the condition, but IL‐1β overexpression can further exacerbate inflammatory reactions and even cause multiorgan failure [21]. IL‐12 is an important immune‐activating cytokine produced mainly by macrophages and dendritic cells, and these cells can release both the biologically inactive form of IL‐12 (IL‐12 p40) and the active form (IL‐12 p70). IL‐12 p70, a heterodimer composed of p35 and p40, is a proinflammatory cytokine capable of inducing the response of Th1 cells, cytotoxic CD8 + T cells, and NK cells. IL‐12 p70 also promotes IL‐2‐dependent T‐cell proliferation and activates CD25 expression on CD8 + T cells [22]. IL‐12 p70 is critical for the immune response against tumors, intracellular parasites, fungi, bacteria, and viruses. IL‐17 is a characteristic cytokine produced by Th17 cells and has strong proinflammatory effects. IL‐17 can stimulate human fibroblasts to express intercellular adhesion molecule‐1 [23], recruit neutrophils and macrophages to infiltrate tissues, induce human endothelial cells and macrophages to secrete IL‐6, IL‐8, prostaglandin E2, and other proinflammatory cytokines, mediate inflammation, and promote the occurrence and development of diseases [24, 25]. IL‐6 is a prototypical cytokine with pleiotropic activity that contributes to maintaining homeostasis [26]; excessive IL‐6 levels and uncontrolled IL‐6 receptor signaling were common in critical COVID‐19 patients [27].

Several studies have evaluated bacterial coinfections in respiratory and blood specimens of hospitalized COVID‐19 patients [28, 29]. The most frequently isolated bacterial species in COVID‐19 patients were *Staphylococcus aureus, Streptococcus pneumoniae, Haemophilus influenzae,* and *K. pneumonia* [30]. In the presented study, the top three bacterial species were *A. baumannii*, *K. pneumonia,* and *P. aeruginosa* in respiratory and blood specimens. Further, carbapenem‐resistant *A. baumannii*, *K. pneumonia,* and *P. aeruginosa* were common antibiotic‐resistant phenotypes. In this study, bacterial detection and culture used deep sputum or BALF samples, which may avoid the influence of colonizing bacteria on the conclusions of this article. Besides, cytokines levels between single and multiple bacterial coinfections were compared, and only IL‐6 showed statistical significance, which may be affected by factors such as small sample size and individual differences of patients.

This study also has some limitations. Vaccination was not included in the baseline data, which was an important factor for the treatment and prognosis of COVID‐19 patients. Besides, this study did not continuously monitor inflammatory markers for individual patients to explore their impact on the treatment of individual disease progression. Further, serial follow‐up work was not systematic enough to obtain more detailed patient outcome data.

## 5 Conclusions

The results showed that serum levels of PCT, CRP, IL‐1β, IL‐2, IL‐5, IL‐6, IL‐8, IL‐12p70, IL‐17, and TNF‐α were higher in COVID‐19 patients with bacterial coinfection than in the bacteria‐negative group, suggesting that the above markers may be involved in the development of bacteria coinfection in COVID‐19 patients. Detection of these cytokines has clinical significance in the management of COVID‐19 patients including diagnosis of inflammatory storm and early warning of sepsis. Monitoring the changes in their levels can be used as a helpful way to evaluate bacterial coinfection in COVID‐19 patients.

## Author Contributions

**Qingqing Bi:** conceptualization, data curation, formal analysis, writing–original draft. **Jie Zhu:** investigation, methodology, resources. **Jinju Zheng:** software, validation, visualization. **Qingyun Xu:** formal analysis, methodology, software. **Juan Chen:** methodology, software. **Lei Zhang:** formal analysis, supervision. **Xiaofeng Mu:** supervision, validation, writing–review and editing.

## Ethics Statement

The study protocol was approved by the Ethical Committee in Affiliated Qingdao Central Hospital, Qingdao University (ID: JS202015101). The approval included permission to access the inpatient medical records and the waiver of informed consent of the patients since all the data were collected retrospectively.

## Conflicts of Interest

The authors declare no conflicts of interest.

## Supporting information

Supporting information.

## Data Availability

The data sets used during the current study are available from the corresponding author upon reasonable request.
