# Supplementary material for: Blood Inflammatory Markers and Cytokines in COVID‐19 Patients With Bacterial Coinfections
Source: Immun Inflamm Dis. 2024 Dec 18;12(12):e70105. doi: 10.1002/iid3.70105 (PMC11653711; doi:10.1002/iid3.70105)
Supplement: Supplementary file 1 — Supporting information. [file IID3-12-e70105-s001.docx]

**Table S1 Antibiotic resistance in bacterial coinfection group**

| Bacteria | Counts | Antibiotic resistance bacteria | Counts | Rate of antibiotic resistance bacteria |
| --- | --- | --- | --- | --- |
| *Acinetobacter baumannii* | 60 | Carbapenem resistant *A.baumannii* | 29 | 48.33% |
| *Klebsiella pneumonia* | 44 | Carbapenem resistant *K.pneumonia* | 5 | 11.36% |
| *Pseudomonas aeruginosa* | 32 | Carbapenem resistant *P.aeruginosa* | 5 | 15.63% |
| *Haemophilus influenzae* | 29 | Ampicillin resistant *H.influenzae* | 18 | 62.07% |
| *Stenotrophomonas maltophilia* | 19 | Levofloxacin resistant *S.maltophilia* | 2 | 10.53% |
| *Staphylococcus aureus* | 29 | *MRSA* | 12 | 41.38% |
| *Escherichia coli* | 11 | Carbapenem resistant *E.coli* | 1 | 9.09% |
| *Streptococcus pneumoniae* | 7 | - | 0 |  |
